# Supplementary material for: Surveillance of Vermont wildlife in 2021–2022 reveals no detected SARS-CoV-2 viral RNA
Source: Sci Rep. 2023 Sep 6;13:14683. doi: 10.1038/s41598-023-39232-0 (PMC10482933; doi:10.1038/s41598-023-39232-0)
Supplement: Supplementary file 1 — Supplementary Information 1. [file 41598_2023_39232_MOESM1_ESM.docx]

**Supplemental Figures**

**Supplemental Figure 1.** Geographic distribution of 2021 sample collection by species. Graphs were generated using QGIS version 3.28.2 (Firenze) https://qgis.org/.

.


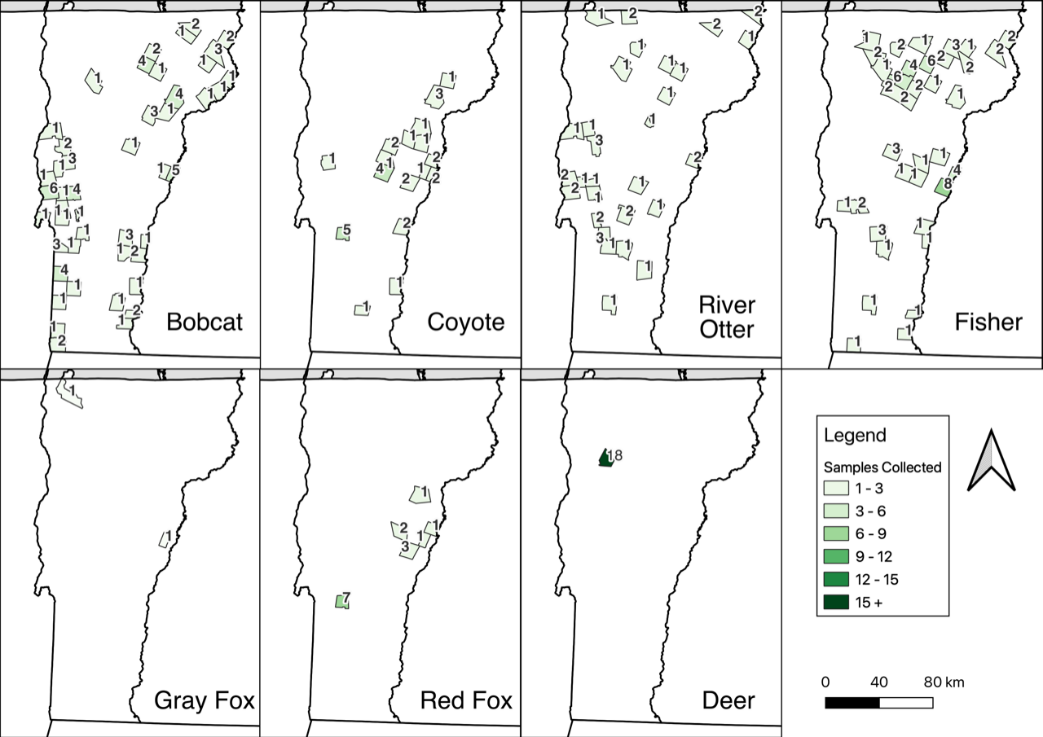


**Supplemental Figure 2: Power to Detect a Given Prevalence.** Using the binomial distribution for 472 trials (the number of deer samples collected in the 2022 hunting season), we calculated the probability of at least one success (SARS-CoV-2 detection) as a function of the unknown underlying SARS-CoV-2 prevalence (percent positivity).
